# Differential immune responses of C57BL/6 mice to infection by *Salmonella enterica* serovar Typhimurium strain SL1344, CVCC541 and CMCC50115

**DOI:** 10.1080/21505594.2019.1597496

**Published:** 2019-04-02

**Authors:** Shao Wei, Jianwei Huang, Zhexi Liu, Mengyao Wang, Bingkun Zhang, Zhengxing Lian, Yuming Guo, Hongbing Han

**Affiliations:** a State Key Laboratory of Animal Nutrition, College of Animal Science and Technology, China Agricultural University, Beijing, China; b Beijing Key Laboratory of Animal Genetic Improvement, College of Animal Science and Technology, China Agricultural University, Beijing, China; c National Engineering Laboratory for Animal Breeding, College of Animal Science and Technology, China Agricultural University, Beijing, China; d Key Laboratory of Animal Genetics, Breeding and Reproduction of the Ministry of Agriculture and Rural Affairs, College of Animal Science and Technology, China Agricultural University, Beijing, China

**Keywords:** *Salmonella enterica* serovar Typhimurium, mice, virulence, immune response

## Abstract

With a broad range of hosts, *Salmonella enterica* serovar Typhimurium (*S*. Typhimurium) is the major cause of gastroenteritis in human beings and systemic disease in susceptible mice strains. However, different *S*. Typhimurium strains differ in regard to virulence and host adaptation. Here, C57BL/6 mice were infected, respectively, with different *S*. Typhimurium strains SL1344 (calf), CVCC541 (chicken) and CMCC50115 (mutton) to determine their virulence and host immune responses. It was found that mice were less susceptible to infection by *S*. Typhimurium CVCC541 and CMCC50115 strains, with lower lethality and decreased bacterial burden in liver and spleen. Besides, *S*. Typhimurium strains CVCC541 and CMCC50115 enhanced host innate immune responses by increased frequencies of macrophages and neutrophils 3 days after infection. But SL1344 strain evaded immune response by inducing apoptosis of macrophages. Moreover, CVCC541 could elicit adaptive immune responses of host 11 days after infection upon examination of the proliferation and activation of CD4^+^ T cells. In addition, 125 and 138 unique mutant coding genes, respectively, in *S*. Typhimurium strains CVCC541 and CMCC50115 and 78 shared mutant coding genes were annotated by genomic alignment to SL1344 genome and the signal pathways involving these genes were further analyzed. The acquired results indicate that different original *S*. Typhimurium strains show differential virulence and may induce diverse immune responses in the same host infected.

## Introduction

*Salmonella enterica*, composed of distinct serovars based on surface antigens, are capable of infecting a wide range of hosts [1], while *S. enterica* serovar Typhimurium (*S*. Typhimurium) exists as the most prototypical serovar associated with human and animal disease. Though one of the major causes of gastroenteritis, *S*. Typhimurium is rare by bloodstream infection in human. However, some variant *S*. Typhimurium strains such as ST313, showing genetic differences compared to other *S*. Typhimurium strains, might cause bloodstream infection characterized by fever [2–4]. *S*. Typhimurium can induce differential responses in the same hosts, which can be related to virulence factors of different strains and various host immune responses. Thus, it is vital to study the interplay between hosts and different *S*. Typhimurium strains to better understand the etiology of new pathogens.

The interaction between pathogens and host defenses is coordinated by the innate and adaptive immune system. The innate immune is efficient in host defense against *S*. Typhimurium replication by rapidly increased macrophages and neutrophils in infected tissues at early phase of infection. But the clearance of invading bacteria depends largely on the activating adaptive immunity of hosts, playing a particularly important role in the later stages of *S*. Typhimurium infection [5,6]. Activated CD4^+^ T cells would undergo clonal expansion and differentiate into effector cells that can secret various cytokines to mediate microbial clearance. Previous study suggested that *Salmonella*-specific class-I-restricted CD8^+^ T cells also took part in the protection during primary infection [7].

As we know *S*. Typhimurium, including a series of variants, enjoys a range of various hosts and has shown a differential degree of host adaptation [1]. In particular, *S*. Typhimurium SL1344 strain, the histidine auxotroph of ancestral strain ST4/74 isolated from calf, is known as a strongly virulent strain [8]. In this study, two *S*. Typhimurium strains, CVCC541 and CMCC50115 (ATCC13311) originally isolated, respectively, from chicken and mutton [9,10], were used to infect C57BL/6 mice in order to investigate the virulence and immune activation of these strains in mice at various stages post infection. It was found that SL1344 strain, more virulent than both CVCC541 and CMCC50115 strains, were capable of eliciting strong proinflammatory cytokines in serum of mice and induced apoptosis of macrophage in spleen at the early phase of infection. At 11 days after infection, only CVCC541 strain triggered host adaptive immune response. In addition, the differences in mutant conding genes among three strains were also analyzed.

## Results

### *Determination of the virulence of* S. *Typhimurium CVCC541, CMCC50115 and SL1344 strains in mice*

**Figure 1.**
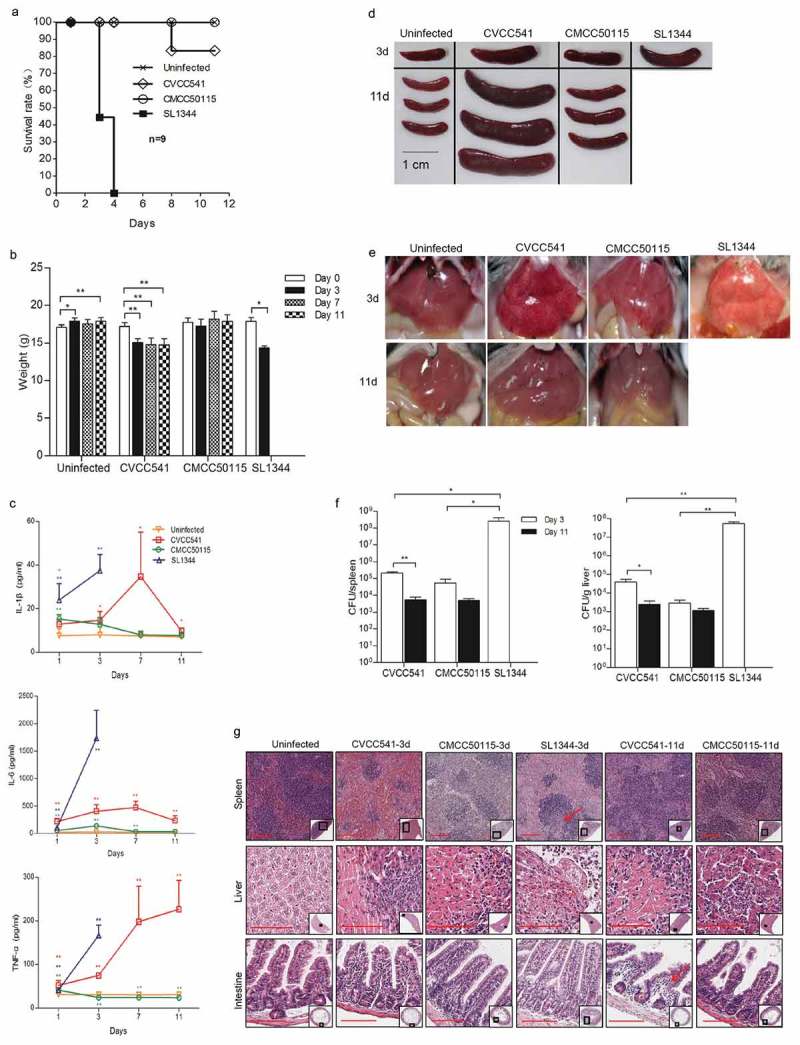
*S*. Typhimurium CVCC541 and CMCC50115 strains are less virulent strains compared to SL1344 strains. C57BL/6 mice were infected intravenously with *S*. Typhimurium CVCC541, CMCC50115 and SL1344 strain (2.5 × 10^5^ CFU) for the indicated days. (a) Survival rate of C57BL/6 mice. (b) Body weight of mice infected by three *S*. Typhimurium strains. (c) Cytokine levels of TNF-α, IL-6, IL-1β in serum of mice by ELISA (n = 4). (d, e) Gross anatomy of spleen (d) and liver (e). (f) Bacterial loads in spleen and liver (n = 3). (g) Hematoxylin and eosin-stained spleen, liver, and intestine sections (Scale bar = 100 μm) of mice at 3 days and 11 days post infection (*p < 0.05, **p < 0.01).

C57BL/6 mice were infected intravenously with 2.5 × 10^5^ CFU of *S*. Typhimurium strains SL1344, CVCC541 and CMCC50115, respectively. At 3 days post-infection, mice infected by SL1344 strain showed high lethality and marked loss of weight, while mice challenged with CVCC541 and CMCC50115 strains displayed significantly improved survival after 11 days (Figure 1a,b). The inflammatory kinetics of serum in mice at 1, 3, 7 and 11 days after infection by SL1344, CVCC541, and CMCC50115 showed that SL1344 induced significantly high levels of IL-1β, IL-6 and TNFα at 1 and 3 days (Figure 1c). In contrast, the release of IL-1β and IL-6 in CVCC541-infected mice was increased significantly at 1, 3 and 7 days but declined at 11 days (Figure 1c), while the expression of TNFα was increased well into 11 days; the secretion of IL-1β and IL-6 in CMCC50115-infected mice, after slight increasing at 1 and 3 days, relapsed to normal levels at 7 days, with the TNFα level starting to decrease after 3 days post infection (Figure 1c). Indeed, mice infected with CVCC541 showed similar spleen size as those infected by SL1344 at 3 days post-infection (Figure 1d) and the size continued enlarging in CVCC541-infected mice at 11 days (Figure 1d). Meanwhile, bleeding spots were observed in the liver of mice infected by CVCC541 and CMCC50115 at 3 and 11 days post-infection (Figure 1e). In addition, the bacterial loads and damage in tissues of mice infected by three *S*. Typhimurium strains were evaluated. The results showed that SL1344-infected mice carried significantly higher bacterial burdens than CVCC541- and CMCC50115-infected mice at 3 days after infection. Compared with that of CMCC50115-infected mice, slightly higher number of tissue bacteria was found in CVCC541-infected mice, although there existed no significant differences (Figure 1f). Yet 11 days after infection, the number of *S*. Typhimurium CVCC541 in spleen and liver was observed to drop significantly (Figure 1f).

Histological detection showed severe splenic disruption of white pulp and necrosis in mice infected with SL1344 and intense congestion of red pulp in mice infected with CVCC541 after 3 days of infection, while the spleens of CVCC541-infected mice were characterized by mounting number of lymphocytes at 11 days post-infection (Figure 1g). In contrast, CMCC50115-infected mice displayed slightly enlarged spleens but no salient pathological damage at both 3 and 11 days post-infection (Figure 1g). In fact, focal necrosis accompanied by mounting number of inflammatory cell infiltration was observed in the livers of all infected mice especially SL1344-infected mice at 3 or 11 days (Figure 1g). Besides, the analysis of intestine sections also showed submucosal edema in CVCC541-infected mice after 11 days (Figure 1g). Collectively, these results indicated that *S*. Typhimurium CVCC541 and CMCC50115 were significantly less virulent compared to SL1344 and *S*. Typhimurium CVCC541 could promote significantly proliferation of lymphocytes compared to CMCC50115.

### *Differential host innate immune responses to* S. *Typhimurium CVCC541, CMCC50115, and SL1344 infection at the early stage of infection*

**Figure 2.**
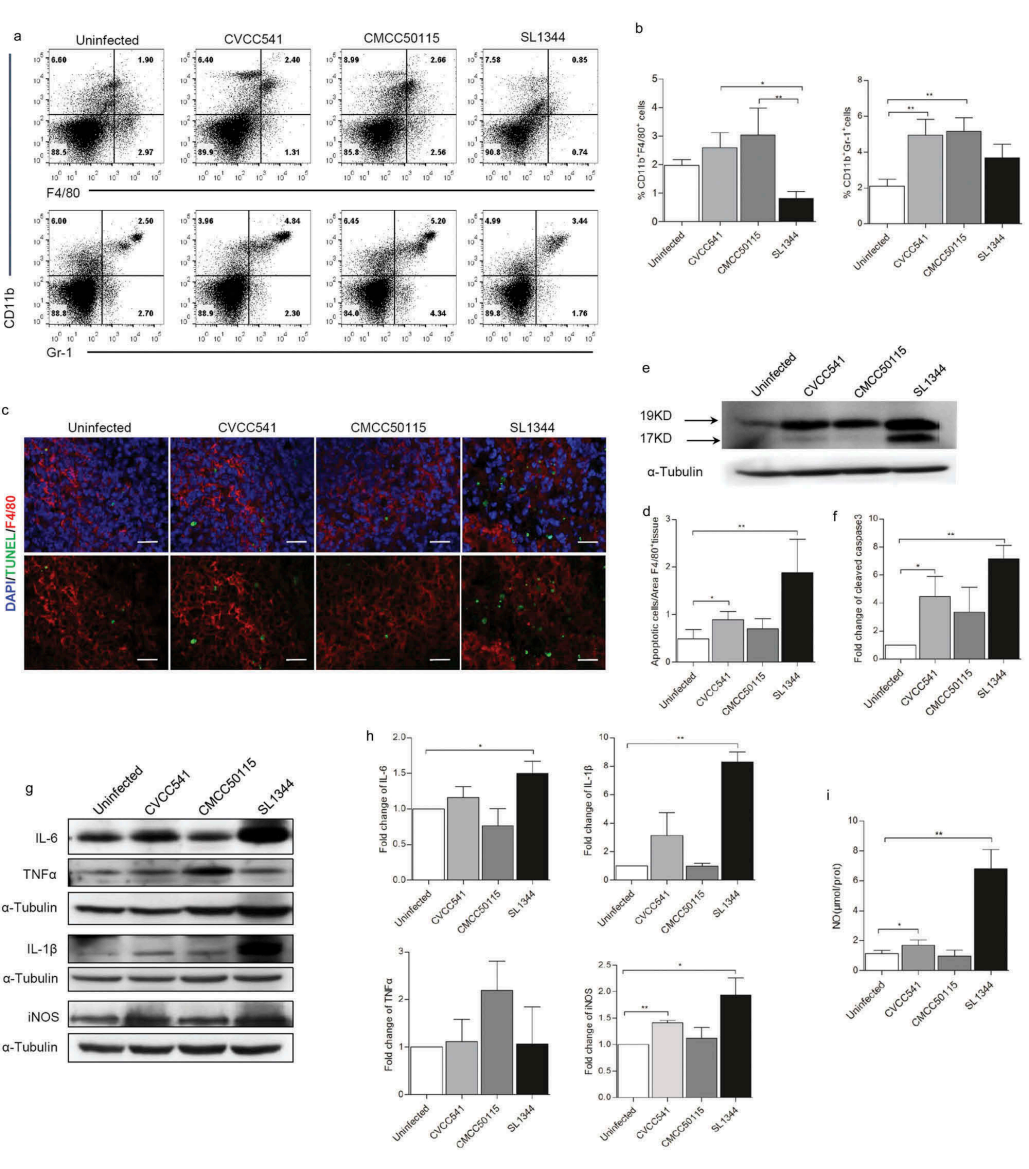
*S*. Typhimurium CVCC541 and CMCC50115 strains induced innate immunity of mice. C57BL/6 mice were infected intravenously by *S*. Typhimurium CVCC541, CMCC50115, and SL1344 strain, respectively, and killed at 3 days after infection. (a) Flow cytometry of macrophages (F4/80^+^CD11b^+^) and neutrophils (Ly6G^+^CD11b^+^) in the spleens of mice infected by *S*. Typhimurium. (b) Quantification of positive cells (n = 3). (c) The detection F4/80^+^ and TUNEL^+^ in frozen sections of spleens (red, anti-F4/80; green, TUNEL; blue, Hoechest33342), Scale bar = 20 μm. (d) Quantification of TUNEL^+^ cells per area F4/80^+^ splenic tissue. (e) Immunoblot analysis of cleaved caspase3 in spleens. (f) Quantification of total densitometry of 17KD and 19KD (n = 3). (g) Immunoblot analysis of cytokine secretion in splenic cells (TNF-α, IL-6, IL-1β,iNOS) at 3 days after infection. (h) Quantification of relative densitometry (The fold change = the densitometry of bands in indicated S. Typhimurium infected mice relative to uninfected mice) (n = 3). (i) The expression of nitric oxide (NO) in spleen homogenates at 3 days. (*p < 0.05, **p < 0.01).

To further investigate host innate immune responses to infection by *S*. Typhimurium strains SL1344, CVCC541 and CMCC50115 at 3 days post-infection. The numbers of macrophages (CD11b^+^F4/80^+^) and neutrophils (Ly6G^+^CD11b^+^) in spleens of infected mice were first detected by flow cytometry. There were significantly increased frequencies of CD11b^+^F4/80^+^ cells in both CVCC541- and CMCC50115-infected mice compared with the SL1344-infected mice. But the frequencies in all infected groups were proven to be insignificantly different from the uninfected controls (Figure 2a,b). It was also noted that the percentages of CD11b^+^F4/80^+^ cells in SL1344-infected mice were lower than that of the uninfected controls, although no statistical differences existed. Furthermore, the frequencies of Ly6G^+^CD11b^+^ cells in CVCC541- and CMCC50115-infected mice were significantly higher than the uninfected mice (Figure 2a,b), but were not significantly different in SL1344-infected spleens. To determine whether *S*. Typhimurium SL1344-induced decrease of macrophages in spleen was associated with apoptosis, the number of TUNEL^+^ splenic macrophages by immunofluorescence staining and the expression of caspase3-cleavage pattern in spleens by immunoblotting were detected. The results revealed that SL1344 prominently enhanced levels of cleaved caspase3 and TUNEL^+^F4/80^+^ population (Figure 2c-f). Thus, it could be concluded that SL1344-induced decrease of macrophages in the spleen of infected mice was probably realized through cell apoptosis.

Furthermore, the level of inflammatory cytokines in spleens was detected by Western Blot. As shown in Figure 2g,h, CMCC50115 induced higher levels of TNFα in comparison with the uninfected group, but CVCC541 and SL1344 strains enhanced expression of IL-6, IL-1β and inducible nitric oxide synthase (iNOS), being responsible for the release of NO. Consistently, significantly increased production of NO at 3 days in spleens of mice infected with CVCC541 and SL1344 was measured (Figure 2i). These results suggested that hosts controlled CVCC541, CMCC50115 and SL1344 strains by the production of different levels of cytokines, and CVCC541 and CMCC50115 strains triggered host innate immunity by the increased frequencies of macrophages and neutrophils.

### *Distinction of T cells responses of mice to* S. *Typhimurium CVCC541 and CMCC50115 at later stage of infection*

**Figure 3.**
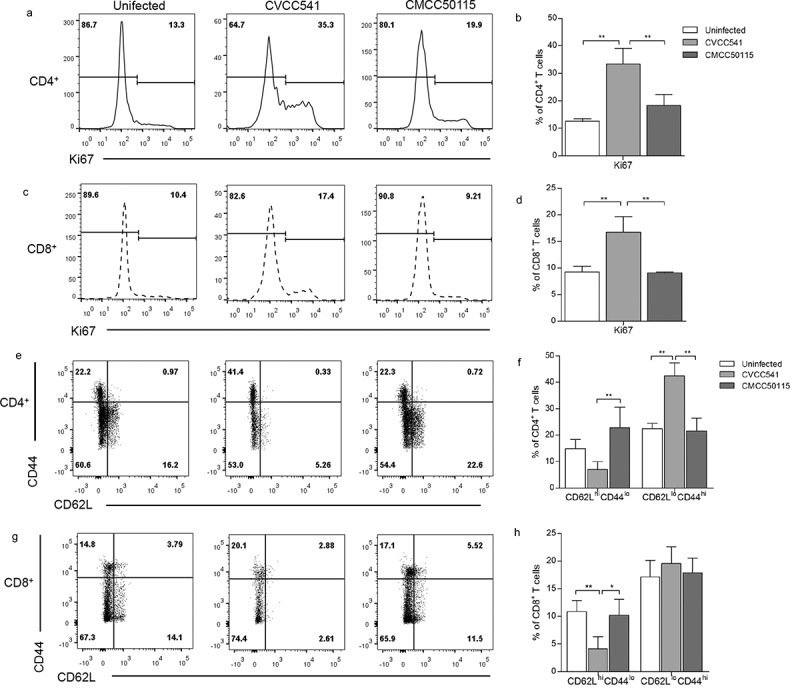
*S*. Typhimurium CVCC541 primed the proliferation and activation of T cells. Mice were infected intravenously by *S*. Typhimurium CVCC541 and CMCC50115 and killed after 11 days of infection. (a-d) The expression of nuclear protein Ki67 in CD4^+^ T cells (a, b) and CD8^+^ T cells (c, d). (e-h) The percentage of CD44^hi^CD62L^lo^ cells in CD4^+^ T cells (e, f) and CD8^+^ T cells (g, h) (n = 4) (*p < 0.05, **p < 0.01).

The T cells proliferation and activation in mice challenged with *S*. Typhimurium strains CVCC541 and CMCC50115 at 11 days after infection were compared in our research. The proliferation of T cells was assessed by the expression of proliferation-associated protein Ki67. In naïve mice, 12% of CD4^+^ T cells and 9% CD8^+^ T cells were labeled by Ki67, but more than 30% of CD4^+^ T cells and 16% of CD8^+^ T cells expressed Ki67 following CVCC541 infection. In comparison, no significant increase of Ki67^+^ in CD4^+^ and CD8^+^ T cells was observed in mice after CMCC50115 infection (Figure 3a-d). Since CD44^low^ and CD62L^high^ are expressed on the surface of naïve T cells, so the expression of CD44 would increase as that of CD62L declines once T cells get activated. Statistically, there existed 20% increased expressions in the CD4^+^CD62L^lo^CD44^hi^ cells of CVCC541-infected mice in comparison with control mice, but no similar increase was observed in CMCC50115-infected mice. Yet, statistical differences were observed in regard to the frequency of CD8^+^CD62L^lo^CD44^hi^ T cells in mice infected with the two *S*. Typhimurium strains as compared to uninfected controls (Figure 3e-h). Taken together, these data suggested that CVCC541, but not CMCC50115, could contribute to host adaptive immunity at 11 days after infection.

### *Comparisons of activation of antigen-presenting cells between* S. *Typhimurium CVCC541 and CMCC50115 at later stage of infection*

**Figure 4.**
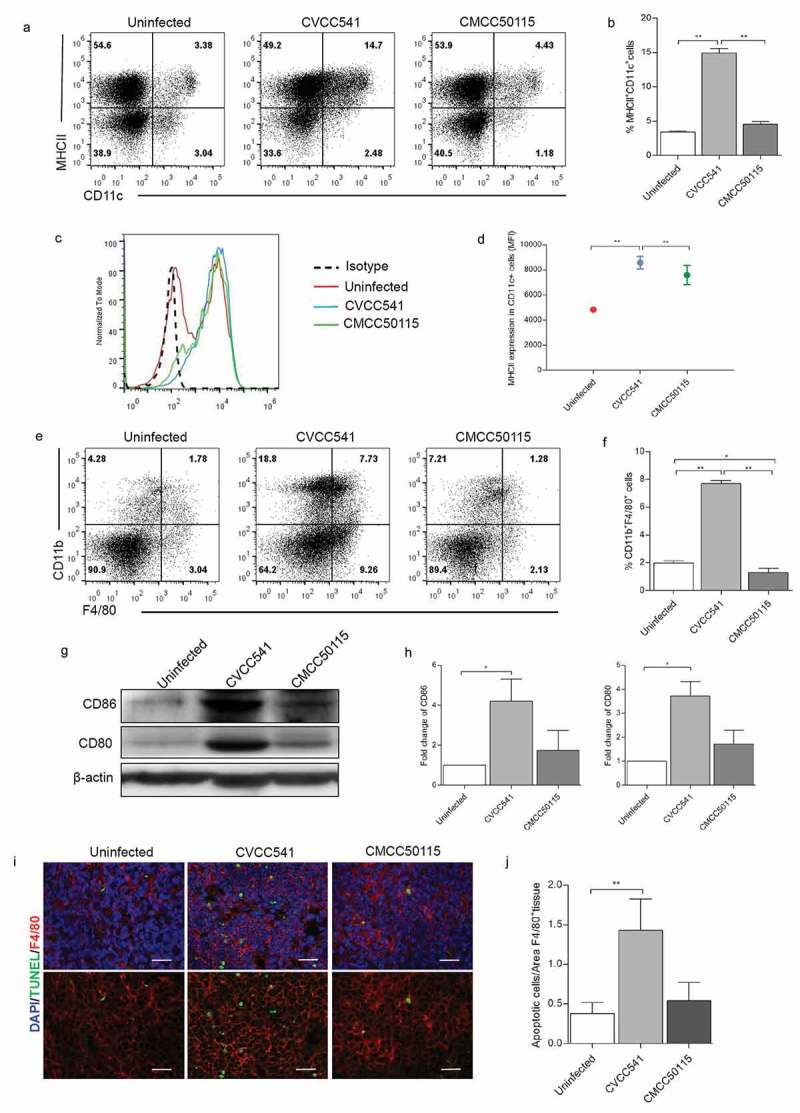
*S*. Typhimurium CVCC541 enhanced the recruitment and stimulation of dendritic cells and macrophages in the spleen at 11 days after infection. (a, b) The percentages of dendritic cells (CD11c^+^MHCII^+^) in the spleen. (c, d) The mean fluorescence intensity of MHCII in CD11c^+^ splenic cells. (e, f) The frequency of macrophages (F4/80^+^CD11b^+^) of spleens (n = 3). (g) The analysis of costimulator CD80 and CD86 in spleen by Western Blot (n = 3). (h) The staining of F4/80 and TUNEL in frozen splenic sections (red, anti-F4/80; green, TUNEL; blue, Hoechest33342), scale bar = 20 μm (*p < 0.05, **p < 0.01).

To further confirm the factors causing CVCC54-induced T cells activation, the major population of antigen-presenting cells (APCs) were analyzed by Flow cytometry. The results showed that approximately 15% DCs (CD11c^+^MHCII^+^) in CVCC541 stimulated-spleens, which was significantly higher than those in both uninfected spleens (3%) and CMCC50115-infected spleens (4%) at 11 days after injection (Figure 4a,b). Moreover, CVCC541 also noticeably enhanced the mean fluorescence intensity of MHCII in CD11c^+^ cells (Figure 4c,d). Besides, compared with uninfected or CMCC50115-infected mice, significant increase of macrophages (CD11b^+^F4/80^+^) was also observed in CVCC541-infected mice (Figure 4e,f). In addition, the activation of APCs was revealed by the enhanced levels of co-stimulator CD80 and CD86 in the spleens of CVCC541-infected mice (Figure 4g,h). Previous study had shown that the activation of T was closely related to the apoptotic macrophages containing bacteria [11]. In this study, more TUNEL^+^F4/80^+^ cells were observed in the spleens of CVCC541-infected mice, and they might also play a part in the activation of T cells (Figure 4i,j). In summary, above results suggested that the activation of T cells in CVCC541-infected mice was mediated by the recruitment of APCs and the apoptosis of macrophages.

### *The genetic diversity of* S. *Typhimurium SL1344, CVCC541 and CMCC50115*

**Figure 5.**
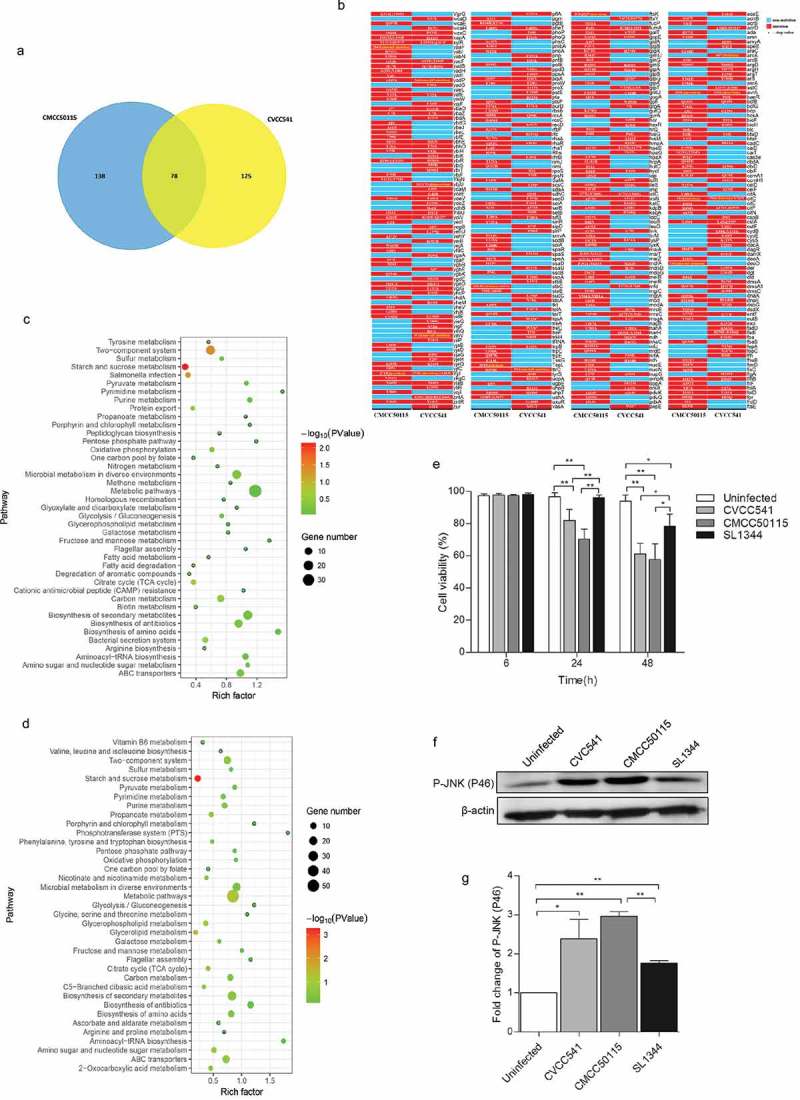
The genomic differences among *S*. Typhimurium CVCC541, CMCC50115 and SL1344 strains. The genomes of CVCC541 and CMCC50115 were analyzed when the genome of SL1344 strain was regarded as the reference sequences. (a) The number of unique and shared mutant protein-coding genes between *S*. Typhimurium CVCC541 and CMCC50115. (b) The mutant sites of all mutant genes were listed (Red box, mutagenesis; Blue box, no mutation). (c, d) Partial metabolic pathway involving mutant protein-coding genes in CVCC541 (c) and CMCC50115 (d). (e) Viability of IEC cells infected by different *S. Typhimurium* strains by measuring lactate dehydrogenase (LDH) activity. (f) Immunoblotting analysis of p-JNK in IEC-6 cells infected with *S. Typhimurium* strains. (g) The quantification of relative densitometry from (f) at 46KDa (*p < 0.05, **p < 0.01).

After investigation into the host immune responses against *S*. Typhimurium CVCC541, CMCC50115, and SL1344 infection at various time points, the genomic differences of the two *S*. Typhimurium strains were also analyzed by using SL1344 as genomic references (NCBI Reference Sequence: NC_016810.1). The results revealed 125 and 138 mutant protein-coding genes, respectively, in CVCC541 and CMCC50115 and 78 shared mutant protein-coding genes when compared to SL1344 (Figure 5a,b). *S*. Typhimurium infection is conducted by Type III secretion system (T3SS) encoded by the pathogenicity islands SPI-1 and SPI-2. Hence, SPI-1 T3SS and SPI-2 T3SS associated genes of *S*. Typhimurium CVCC541 and CMCC50115 were evaluated by comparative genomic analysis. It was found that there existed three mutant SPI-1 genes (avrA, SipD, SpaS) and two SPI-2 genes (SsaU, SifB) in CVCC541, three mutant SPI-1 genes (avrA, SteB, SpaR) and one SPI-2 gene (SifB) in CMCC50115 (Figure 5b), with the SNP and amino acid changes of those genes as shown in Table S1.

In addition, the signal pathways of mutant genes involved in both CVCC541 and CMCC50115 infection were analyzed (Figure 5c,d, Table S2). As a result, there were 16 and 14 mutant protein-coding genes in CVCC541 and CMCC50115 belonging to two-component system, respectively, which regulate *Salmonella* virulence, with the changes of SNP and relative amino acid of important genes (PhoP, PhoQ, and OMPR) as shown in (Table S1).

Former studies have shown that *Salmonella* avrA inhibits the apoptosis of epithelial cells and the activation of Phospho-JNK [12,13]. Here, rat primary epithelial cells (IEC-6) were infected with *S*. Typhimurium strains CVCC541, CMCC50115 and SL1344 for 6, 24 and 48 h, it was found that CVCC541, CMCC50115 strains carrying avrA mutant gene significantly promoted the apoptosis of IEC-6 at 24 and 48 h after infection as compared to uninfected and SL1344-infected cells (Figure 5e). Furthermore, CVCC541 and CMCC50115 strains enhanced the expression of p-JNK at 2 h (Figure 5f,g). Taken together, these results showed that genetic differences existed among *S*. Typhimurium strains CVCC541, CMCC50115, and SL1344, which would modulate bacterial virulence and facilitate different host immune responses. However, the question whether such differences are mediated by a single gene or their additive effect remains undetermined.

## Discussion

*S. enterica* serotype Typhimurium SL1344, causing the death of mice at the low dose, is the highly virulent strain [14]. In contrast, mice infected by CVCC541 and CMCC50115 showed higher survival rate and lower bacterial burden. Moreover, the upregulated levels of proinflammatory cytokines IL-1β, IL-6 and TNFα in the serum of mice infected by SL1344 manifested their stronger resistance to bacterial infection, which implied that the SL1344 had posed higher virulence to hosts. In addition to splenic disruption in CVCC541 and SL1344-infected mice, focal necrosis and inflammation were observed in the livers of all infected mice, and those infected by SL1344 in particular. However, there existed no obvious small intestinal pathology in systemically infected mice. Additionally, we observed similar splenomegaly in CVCC541 and SL1344 infected mice at 3 days after infection and spleens in mice infected by CVCC541 strain severely enlarged at 11 days. By the assessment of bacterial burden in spleen and histological analysis of splenic sections, the different mechanisms of splenomegaly were shown. At 3 days after infection, there was increased number of SL1344, which led to splenomegaly and accelerated the structural damage of spleens. By contrast, the spleens of mice infected with CVCC541 strain were loaded with lower bacterial counts at 3 days post-infection. Thus, the splenomegaly resulted from red pulp congestion. At 11 days following infection, the spleens severely enlarged accompanied by the clearance of bacteria, which were consistent with previous studies which suggested *S*. Typhimurium stimulated splenomegaly and expanded spleen cells in non-lethal mice, which aimed to clearing bacteria [15]. Thus, we concluded different *S*. Typhimurium strains could induce tissue lesions by different pathological mechanisms.

The immune cells are vital to pathogen control during the early stages of infection. In our study it has been shown that there was slighter bacterial burden in CVCC54- and CMCC50115-infected mice, whose increased macrophages and neutrophils suggested that the bacterial control was mediated by these innate immune cells. In contrast, SL1344 infection was observed to have impaired the frequencies of macrophages in this research. Being strongly virulent pathogen, SL1344 was reported to enhance the apoptosis of macrophages [14]. Consistent with that, elevated apoptotic F4/80^+^ macrophages were observed in SL1344 infected spleen. Furthermore, higher levels of IL-6, IL-1β and iNOS were detected in the spleen of mice infected by *S*. Typhimurium SL1344, which can be explained that strongly virulent SL1344 elicited intense host immunologic tissue response to resist bacterial invade. The high virulence of SL1344 led to damage of tissue and host finally submitted to increased dissemination of the bacteria. Above these findings suggested the importance of innate immune cells and proinflammatory cytokines in impairing the infection of attenuated virulent strains at the early stage.

Furthermore, it was discovered that *S*. Typhimurium CVCC541 promoted the proliferation and activation of CD4^+^ and CD8^+^ T cells at day 11 after infection. With DCs identified as the most efficient APCs in activating naïve T cells and macrophages also participate in adaptive immune responses although they are not as efficient as DCs [16]. In this study, increased frequencies of CD11c^+^MHCII^+^ DCs and CD11b^+^F4/80^+^ macrophages and higher levels of CD80 and CD86 were evaluated in CVCC541-infected mice, which suggested that T cells activation was closely related to the recruitment of activated APCs. In addition, previous report also indicated that bystander DCs were capable of internalizing apoptotic macrophages with bacterial antigen caused by *S*. Typhimurium and presenting epitopes on MHC-I and MHC-II, which can induce the activation of CD4^+^ and CD8^+^ T cells [11]. Hence, it can be summarized that greater number of apoptotic macrophages in CVCC541-infected spleen section might account for the enhanced stimulation of T cells.

The results showed that three strains presented different degrees of host adaptation, which in turn triggered various immune responses. By comparing the differences of genome among three strains, we found that the virulence of *S*. Typhimurium relies to a larger extent on SPI-1 and SPI-2 effector proteins. SPI-1 T3SS effectors function bacterial invasion of epithelial cells [17], while SPI-2 encoded T3SS is responsible for intestinal [18] and disseminated infection and the proliferation within macrophages [19]. Previous study demonstrated that SPI-2 encoded T3SS effectors varies among different *Salmonella serovars* and subtle variations existed in different strains of the same *Salmonella serovars* and determined the virulence of *S. enterica* [20]. In our study, two mutational SPI-2 effector genes (SsaU, SifB) of CVCC541 strain and one gene (SifB) of CMCC50115 strain were found as aligned to SL1344 strain. However, the functions of these genes still remain unclear. The virulence of *S*. enterica is mediated by the complex network of TCSs, with PhoP-PhoQ TCS as the major regulator of *Salmonella* virulence. Controlling the expression of spi/ssa genes which belongs to type three secretion system encoded by the SPI-2 pathogenicity island, PhoP-PhoQ is indispensable for the survival of intra-bacteria by regulating the two-component SsrB/SsrA system. In addition, transcription of ssrB-ssrA inside macrophages also requires the OmpR-EnvZ TCS [21,22]. In our study, mutations in PhoP and PhoQ genes in CVCC541 and mutation in OmpR genes in CMCC50115 were annotated by using SL1344 genome as reference, which partly explained the significantly lighter bacterial burdens in CVCC541 and CMCC50115-infected spleens and livers than those in SL1344-infected counterparts.

Taken all acquired results together, it could be concluded that CVCC541 and CMCC50115, being attenuated *S*. Typhimurium strains, could enhance innate immunity of mice 3 days post infection. However, after 11 days of infection, only CVCC541 strain had initiated adaptive immune response. Furthermore, all these diversities in immune responses can be attributed to their genomic differences existing among the three strains, which eventually modulated the different interaction between pathogen and hosts.

## Materials and methods

### Ethics statement

All experimental animal protocols were approved by the Animal Care and Use Committee at China Agricultural University.

### Bacterial strains

Three strains of *Salmonella enterica* serovar Typhimurium were used in the study, with SL1344 donated by Jilin University, CVCC541 acquired from the Chinese Veterinary Culture Collection Center, and CMCC50115 purchased from the National Center for Medical Culture Collections. The *S*. Typhimurium strain SL1344 was grown at 37 °C overnight in Luria-Bertani (LB) broth supplemented with 50 μg/ml streptomycin, while CVCC541 and CMCC50115 strains cultured simply in LB broth. The bacterial dose was determined on LB agar plates with or without 50 μg/ml streptomycin.

### Mice and infection

Obtained from Beijing Vital River Laboratory Animal Technology Co., Ltd., female C57BL/6 mice were kept under specific-pathogen-free conditions. For experiment, the mice (6–8 weeks) were infected intravenously with three different *S*. Typhimurium strains suspended in 200 µl of PBS (2.5 × 10^5^). Three days and 11 days after infection, spleen, and liver were weighed and homogenized in PBS with appropriate serial dilutions were plated on LB agar. The bacterial burden was confirmed overnight at 37°C.

### Histopathology and immunofluorescence

For histological assessment, the spleen, liver, and intestine were fixed in 4% paraformaldehyde and embedded in paraffin. Three micrometer sections were stained with hematoxylin and eosin (H&E). The entire images were captured by APERIO CS2 (Leica, Germany). For immunofluorescence staining, the spleens were frozen in OCT and cut into 8 μm sections. After being fixed by 4% paraformaldehyde at room temperature (RT) for 10 min, the sections were treated Blocking Solution (Beyotime, China) at RT for 1 h. Then, the samples were probed with the primary antibody Anti-F4/80 (1:150, Abcam, Cambridge, UK) at 4 °C overnight, then incubated with the secondary antibody DyLight 550 donkey anti-rat IgG (H + L) Cross Adsorbed (1:500, Life Technologies, Carlsbad, CA, USA) at RT for 1 h. Cell apoptosis was detected using TUNEL staining kit (KeyGEN BioTECH, Nanjing, China) according to the manufacturer’s instructions. The fluorescence was observed by confocal microscopy. The apoptotic cells of macrophages were quantitatively analyzed (apoptotic cells/F4/80^+^ area) by software ImageJ.

### ELISA experiments

C57BL/6 mice were injected intravenously with 2.5 × 10^5^ S. Typhimurium CVCC541, CMCC50115 and SL1344 strains at 1, 3, 7, 11 d, and levels of IL-1β, IL-6, and TNFα in serum from surviving animals were measured. ELISA kits were from Proteintech (China).

### Nitric oxide assay

Spleen was removed from mice infected by S. Typhimurium CVCC541, CMCC50115 and SL1344 strains for 3d and homogenated in PBS and then protein was quantified by BCA protein assay kit (Thermo Fisher Scientific, USA). The release of nitric oxide (NO) was measured by Nitric Oxide Assay Kit (KeyGEN BioTECH, China) following manufacturer^,^ s protocol.

### Western blotting

The spleens from each group were homogenized and lysed in RIPA buffer (Aidlab Biotechnologies Co Ltd., Beijing, China) supplemented with protease inhibitor PMSF, phosphatase inhibitors (Aidlab Biotechnologies Co Ltd., China) and protease inhibitors (Beyotime Biotechnology, China). For Western blotting, BCA protein assay kit (Thermo Fisher Scientific, USA) was used to measure protein concentrations. After being mixed with 6 × loading buffer and heated at 95°C. The protein (30 μg) was resolved by 8–12% SDS-PAGE and transferred to the PVDF membranes (Millipore, USA). The membranes were blocked with 5% milk in TBS supplemented with 0.1% Tween 20 at RT for 1–2 h and incubated at 4°C for 16 h with the following primary antibodies: TNF-α antibody (Proteintech, China), IL-6 antibody (Proteintech, China), IL-1β (Proteintech, China), β-actin (Proteintech, China), CD80 (Proteintech, China), α-tublin (Beyotime, China), iNOS (abcam, UK), CD86 (Bioss, China), Cleaved caspase3 (Cell Signaling Technology, USA), P-JNK (Abclonal, China). After being washed three times, the membranes were treated with HRP-conjugated secondary antibodies (Zhongshan Biotechnology, China) at RT for 1 h. The blots were visualized using the enhanced chemiluminescence substrate (BioRad, USA) and images were captured by Azure biosystems. All bands detected were within the linear range of detection and then the densitometry of blots was analyzed by ImageJ software.

### Flow cytometry

Single cell suspensions (1 × 10^6^ cells/100 μl) were prepared with dead cells excluded by using Zombie Aqua Fixable Viability Kit (Biolegend, USA). After being washed, cells were resuspended in buffer (PBS containing 5% FBS) and were blocked in Fc block (Anti-mouse CD16/32, Clone 2.4G2, BD, USA) at 4°C for 30 min. Cells were then incubated with following various antibodies for 30 min at 4°C. anti-CD11b (clone M1/70, Biolegend, USA), anti-F4/80 (clone BM8, Biolegend, USA), anti-Ly-6G/Ly-6C (clone RB6-8C5, eBioscience, USA), anti-CD11c (clone N418, Biolegend, USA), anti-I-A/I-E (clone M5/114.15.2, Biolegend, USA); Rat IgG2b,ҝ Isotype Ctrl (Biolegend, USA); anti-CD3 (clone 17A2, Biolegend, USA), Anti-CD4 (clone RM4-5, BD, USA), anti-CD8α (clone 53–6.7, BD, USA), anti-CD44 (clone IM7, BD, USA); anti-CD62L (clone MEL-14, eBioscience, USA), anti-Ki67 (clone SolA15, eBioscience, USA). Ki67 staining was implemented using Foxp3/Transcription Factor Staining Buffer Set (eBioscience, USA) according to manufacturers’ instructions.

### Samples, whole-genome resequencing and alignment

*S*. Typhimurium CVCC541 and CMCC50115 were resequenced on the Illumina HiseqTM 2000 platform and original data had been deposited in public repositories (NCBI, SRA accession: PRJNA515189). Raw resequencing reads were filtered on the basis of chastity score and trimmed on the basis of quality score. The 150-bp paired-end clean reads were then aligned to the reference genome assembly *S*. Typhimurium (SL1344) using bwa-0.7.15. In addition, the paired reads mapped to the exact same position on the reference genome were removed with the function – MarkDuplicates in Picard-tools-1.119 to avoid any influence on variant detection. We finally performed local realignment using GATK 3.6–0-g89b7209 to enhance the alignments in regions of indel polymorphisms.

### Variant discovery and filtering

Variants were called using GATK 3.6–0-g89b7209, and the output was filtered by Vcftools_0.1.13 and Python vcf file parser PyVCF. Variants (SNP/Indel) were then removed if they had two or more alternative alleles, no observations of the alternative allele on either the forward or reverse reads, an overall quality (QUAL) score of <20, a mapping quality (MQ) score of <30. Furthermore, we applied the following proximity filters: when any variant was within 3 bp of another variant, the variant with the lower QUAL score was removed, and when the length of indels were ≥7bp the indels were removed.

### Functional annotation of genetic variants

Identified variants were further classified in gene and intergenic region based on the gene annotation of the reference genome assembly *S*. Typhimurium (SL1344). We selected the variants that were located in genes and made annotation for every variant using a house shell. The signal pathways or metabolic pathways of functional mutant genes were analyzed by comparison to the Kyoto encyclopedia of genes (KEGG) database using DAVID tools (https://david.ncifcrf.gov/).

### Cell infection and cell viability assay

IEC-6 cells (rat small intestine epithelial, China Infrastructure of Cell Line Resource) were cultured in Dulbecco’s Modified Eagle Medium supplemented (Gibco, USA) with 5% FBS (Gibco, USA) and 0.01 mg/ml Insulin. Cells were infected with S. Typhimurium as previously described [23]. In brief, cells were seeded on 96-well and 60 mm plates and infected with S. Typhimurium SL1344, CVCC541 and CMCC50115 strains at a multiplicity of infection of 100. After 45 min of infection, cells were washed in PBS and cultured in fresh medium for additional 20 min. Cells were then incubated in medium containing 50 μg/ml gentamicin for 1 h followed by 5 μg/ml gentamicin and then cells (2 h) were lysed in RIPA buffer (Aidlab Biotechnologies Co Ltd., China) supplemented with protease inhibitor, protease inhibitors and phosphatase inhibitors for the detection of p-JNK by Western Blotting. At 6, 24 and 48 h after infection, cells viability was determined by the measurement of the release of lactate dehydrogenase (LDH) in the medium. LDH kit was from Dojindo, Japan.

### Statistical analysis

Data were presented as mean ± SD. The differences were analyzed by unpaired *t*-test (body weight), paired student’s *t*-test or one-way ANOVA with GraphPad Prism.v5.0 and SPSS (WB). The statistical significance was defined at p < 0.05. Besides, Figure 5a,c was generated by R Software.

## Supplementary Material

Supplemental Material
