# Supplementary material for: Differential immune responses of C57BL/6 mice to infection by Salmonella enterica serovar Typhimurium strain SL1344, CVCC541 and CMCC50115
Source: Virulence. 2019 Apr 2;10(1):248–59. doi: 10.1080/21505594.2019.1597496 (PMC6527021; doi:10.1080/21505594.2019.1597496)
Supplement: Supplemental Material [file kvir-10-01-1597496-s001.docx]

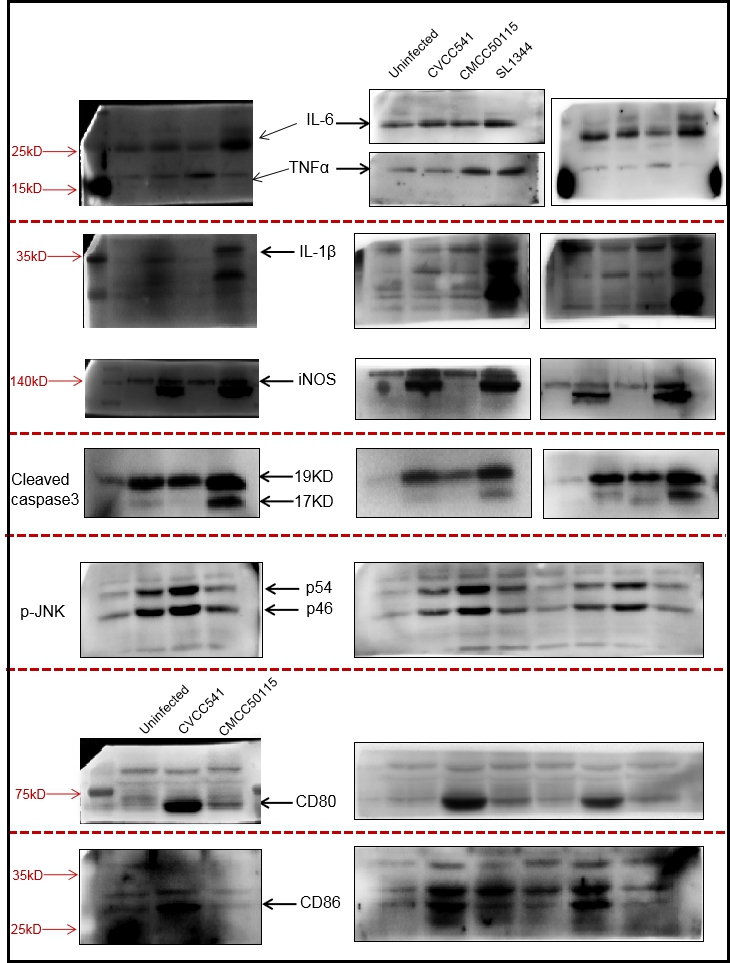


Fig. S1. The entire images for Western blots.

Table S1. The SNP and [amino](D:/Program%20Files/%E6%96%B0%E5%BB%BA%E6%96%87%E4%BB%B6%E5%A4%B9/Youdao/Dict/8.5.1.0/resultui/html/index.html#/javascript:;) [acid](D:/Program%20Files/%E6%96%B0%E5%BB%BA%E6%96%87%E4%BB%B6%E5%A4%B9/Youdao/Dict/8.5.1.0/resultui/html/index.html#/javascript:;) change of mutant protein coding genes

|  | **Gene** | **start** | **end** | **SNP-position** | **Reference** | **Mutation** | **Amino acid (reference)** | **Amino acid (mutation)** |
| --- | --- | --- | --- | --- | --- | --- | --- | --- |
| CVCC541 (SPI1) | avrA | 3032477 | 3033382 | 3032927 | T | TAAA | / | **Insert L (Leucine)** |
|  | sipD | 3049331 | 3050362 | 3049545 | G | T | A (Alanine) | E (Glutamic acid) |
|  | spaS | 3054109 | 3055179 | 3055076 | G | A | T (Threonine) | M (Methionine) |
| CVCC541 (SPI2) | ssaU | 1457283 | 1458341 | 1457952 | C | T | H (Histidine) | Y (Tyrosine) |
|  | sifB | 1649034 | 1649984 | 1649898 | A | G | T (Threonine) | A (Alanine) |
| CMCC50115 (SPI1) | avrA | 3032477 | 3033382 | 3032927 | T | TAAA | / | **Insert L (Leucine)** |
|  | steB | 1676474 | 1676875 | 1676682 | C | T | P (Proline) | L (Leucine) |
|  | spaR | 3055166 | 3055957 | 3055452 | G | A | P (Proline) | L (Leucine) |
| CMCC501115 (SPI2) | sifB | 1649034 | 1649984 | 1649898 | A | G | T (Threonine) | A (Alanine) |
| CVCC541 | phoP | 1275542 | 1276216 | 1275747 | G | A | T (Threonine) | I (Isoleucine) |
|  | phoQ | 1274079 | 1275542 | 1275022 | G | C | T (Threonine) | S(Serine) |
| CMCC50115 | ompR | 3681128 | 3681847 | 3681179 | C | A | Q (Glutamine) | H (Histidine) |

Table S2. Metabolic pathway involving mutant protein coding genes in CVCC541and CMCC50115

|  | **Category** | **Term** | **Count** | **PValue** | **Genes** | **Fold Enrichment** |
| --- | --- | --- | --- | --- | --- | --- |
| **CVCC541** | stm00500 | Starch and sucrose metabolism | 7 | 0.007376723 | AMYA, TREC, TREF, OTSB, GLGX, YAJF, YIHQ | 3.854605263 |
|  | stm02020 | Two-component system | 16 | 0.040920237 | RCSA, TTRC, CITC, UHPT, ARCA, CITF, KDPB, UHPB, CYDB, BAER, NARH, PHOQ, PSTS, PHOP, YEGB, ACRD | 1.688244564 |
|  | stm05132 | Salmonella infection | 5 | 0.053887754 | NRFA, AVRA, SIPD, YGAA, HMPA | 3.388663968 |
|  | stm00020 | Citrate cycle (TCA cycle) | 4 | 0.175987981 | SUCC, ACEE, SDHC, FUMA | 2.710931174 |
|  | stm03070 | Bacterial secretion system | 5 | 0.256103342 | SECD, FTSY, SSAU, SPAS, FFH | 1.915331808 |
|  | stm03060 | Protein export | 3 | 0.289480644 | SECD, FTSY, FFH | 2.782271468 |
|  | stm01200 | Carbon metabolism | 9 | 0.315391148 | SUCC, ACEE, SDHC, YBHE, PTA, FBA, FUMA, KATE, FOLD | 1.379038902 |
|  | stm00190 | Oxidative phosphorylation | 4 | 0.438447333 | CYDB, SDHC, CYOE, NDH | 1.639167687 |
|  | stm01220 | Degradation of aromatic compounds | 2 | 0.471475186 | HPAG, HPAB | 3.203827751 |
|  | stm00071 | Fatty acid degradation | 2 | 0.529552554 | FADD, FADI | 2.710931174 |
|  | stm00670 | One carbon pool by folate | 2 | 0.529552554 | FOLA, FOLD | 2.710931174 |
|  | stm01120 | Microbial metabolism in diverse environments | 15 | 0.531608671 | NRFA, ARGD, TTRC, YBHE, PTA, FUMA, SUCC, ACEE, SDHC, NARH, FBA, HPAG, HPAB, FOLD, FADI | 1.070104411 |
|  | stm00780 | Biotin metabolism | 2 | 0.556176311 | BIOH, BIOF | 2.517293233 |
|  | stm01130 | Biosynthesis of antibiotics | 12 | 0.601784897 | GPH, SUCC, ARGD, ACEE, SDHC, YBHE, FBA, FUMA, KATE, PURD, ARGH, FADI | 1.041638579 |
|  | stm00220 | Arginine biosynthesis | 2 | 0.648560318 | ARGD, ARGH | 1.957894737 |
|  | stm02010 | ABC transporters | 10 | 0.651167261 | PROW, PROX, ARGT, BTUD, LIVK, ARTI, OPPA, FEPC, PSTS, HISM | 1.018557956 |
|  | stm00920 | Sulfur metabolism | 3 | 0.654753485 | DMSA, TTRC, DMSC | 1.355465587 |
|  | stm00010 | Glycolysis / Gluconeogenesis | 3 | 0.654753485 | ACEE, FBA, CELF | 1.355465587 |
|  | stm01212 | Fatty acid metabolism | 2 | 0.687337066 | FADD, FADI | 1.762105263 |
|  | stm00350 | Tyrosine metabolism | 2 | 0.687337066 | HPAG, HPAB | 1.762105263 |
|  | stm00910 | Nitrogen metabolism | 2 | 0.752635312 | NRFA, NARH | 1.468421053 |
|  | stm01110 | Biosynthesis of secondary metabolites | 16 | 0.756303538 | GPH, ARGD, GLPA, YBHE, FUMA, PURD, HISG, TRPB, SUCC, ACEE, SDHC, CYOE, FBA, KATE, ARGH, FADI | 0.927423823 |
|  | stm00970 | Aminoacyl-tRNA biosynthesis | 6 | 0.762222289 | PHET, SELA, GLNS, TYRS, CYSS, ILES | 0.952489331 |
|  | stm00550 | Peptidoglycan biosynthesis | 2 | 0.766727603 | MRCB, DACA | 1.409684211 |
|  | stm03440 | Homologous recombination | 2 | 0.792572231 | DNAE, RECD | 1.305263158 |
|  | stm00052 | Galactose metabolism | 2 | 0.815580585 | GALT, YIHQ | 1.215245009 |
|  | stm00564 | Glycerophospholipid metabolism | 2 | 0.815580585 | GLPA, YBHO | 1.215245009 |
|  | stm00680 | Methane metabolism | 2 | 0.826118838 | PTA, FBA | 1.174736842 |
|  | stm00230 | Purine metabolism | 4 | 0.827305102 | DGT, DNAE, PURD, PNP | 0.903643725 |
|  | stm00620 | Pyruvate metabolism | 3 | 0.834536324 | ACEE, PTA, FUMA | 0.943984962 |
|  | stm00520 | Amino sugar and nucleotide sugar metabolism | 3 | 0.841910562 | GALT, NANA, YAJF | 0.927423823 |
|  | stm00630 | Glyoxylate and dicarboxylate metabolism | 2 | 0.854288417 | GPH, KATE | 1.067942584 |
|  | stm01503 | Cationic antimicrobial peptide (CAMP) resistance | 2 | 0.877935049 | PHOQ, PHOP | 0.978947368 |
|  | stm02040 | Flagellar assembly | 2 | 0.884939997 | FLGA, FLHB | 0.952489331 |
|  | stm00640 | Propanoate metabolism | 2 | 0.884939997 | SUCC, PTA | 0.952489331 |
|  | stm00860 | Porphyrin and chlorophyll metabolism | 2 | 0.897778394 | CBID, CYOE | 0.903643725 |
|  | stm00030 | Pentose phosphate pathway | 2 | 0.914424649 | YBHE, FBA | 0.839097744 |
|  | stm00051 | Fructose and mannose metabolism | 2 | 0.940086976 | FBA, YAJF | 0.734210526 |
|  | stm01100 | Metabolic pathways | 35 | 0.942564961 | PNTB, GPH, DNAE, YBHE, FUMA, BIOF, YIHQ, ACEE, GLNS, FADI, YBHO, CBID, ARGD, TREF, GALT, PTA, FADD, DACA, PURD, HISG, AMYA, TRPB, FOLA, BIOH, SUCC, CYDB, SDHC, GLGX, OTSB, CYOE, FBA, YAJF, ARGH, FOLD | 0.848331282 |
|  | stm01230 | Biosynthesis of amino acids | 5 | 0.944918443 | HISG, TRPB, ARGD, FBA, ARGH | 0.677732794 |
|  | stm00240 | Pyrimidine metabolism | 2 | 0.958110595 | DNAE, PNP | 0.652631579 |

|  | **Category** | **Term** | **Count** | **PValue** | **Genes** | **Fold Enrichment** |
| --- | --- | --- | --- | --- | --- | --- |
| **CMCC50115** | stm00500 | Starch and sucrose metabolism | 9 | 5.11E-04 | GLGB, RFBF, TREA, MALZ, OTSB, GLGX, BCSA, PGM, YAJF | 4.483928571 |
|  | stm00561 | Glycerolipid metabolism | 4 | 0.033973871 | GLPK, GLDA, PLSB | 5.314285714 |
|  | stm01100 | Metabolic pathways | 54 | 0.067501074 | FRDD, GLDA, FUMA, EUTB, GLPK, NUOE, ACNB, PDXA, PGM, PNCA, GLGB, TREA, ARGD, MALZ, NUON, PURD, TRPC, TRPB, NIFJ, TRPE, SUCC, OTSB, GLGX, YAJF, RFBF, USHA, DNAE, YBHE, FBAB, BCSA, BIOF, YADI, SPEA, NAGB, GLNS, SDAA, NRDE, LDCC, YBHO, CBID, CBIF, GALT, PLSB, HISG, FOLA, GLMU, LEUB, DEOD, LEUD, DEOA, FOLD | 1.184201218 |
|  | stm00564 | Glycerophospholipid metabolism | 5 | 0.100915007 | GLPB, GLPA, EUTB, PLSB, YBHO | 2.748768473 |
|  | stm00520 | Amino sugar and nucleotide sugar metabolism | 7 | 0.137907914 | RFBF, GLMU, NAGB, GALT, NANA, PGM, YAJF | 1.957894737 |
|  | stm02010 | ABC transporters | 15 | 0.177977774 | BTUF, YOJI, BTUD, YEJA, FTSE, ARTI, MODA, RBSB, UGPE, PROW, MGLC, LIVK, LIVM, YEHY, FHUB | 1.382328654 |
|  | stm00640 | Propanoate metabolism | 5 | 0.193881275 | SUCC, PDUQ, ACNB, GLDA | 2.154440154 |
|  | stm00020 | Citrate cycle (TCA cycle) | 4 | 0.216303402 | SUCC, FRDD, ACNB, FUMA | 2.452747253 |
|  | stm02020 | Two-component system | 14 | 0.230451973 | GLNG, RCSA, FRDD, DNAA, CITE, TTRC, CITC, UHPT, CITF, KDPB, ARCB, RCSC, OMPR, PGTB | 1.336526946 |
|  | stm01110 | Biosynthesis of secondary metabolites | 23 | 0.242197451 | GLGB, USHA, ARGD, FRDD, GLPB, GLPA, YBHE, FBAB, FUMA, PURD, PLSB, HISG, TRPC, TRPB, TRPE, SUCC, ACNB, LEUB, DEOD, SDAA, LEUD, PGM, LDCC | 1.206203008 |
|  | stm00660 | C5-Branched dibasic acid metabolism | 3 | 0.261718043 | SUCC, LEUB, LEUD | 2.989285714 |
|  | stm01210 | 2-Oxocarboxylic acid metabolism | 4 | 0.267357119 | ARGD, ACNB, LEUB, LEUD | 2.199014778 |
|  | stm00760 | Nicotinate and nicotinamide metabolism | 3 | 0.309066536 | USHA, DEOD, PNCA | 2.657142857 |
|  | stm00230 | Purine metabolism | 7 | 0.355103179 | USHA, DNAE, DEOD, NRDE, PURD, AMN, PGM | 1.430769231 |
|  | stm01230 | Biosynthesis of amino acids | 10 | 0.418442129 | HISG, TRPC, TRPB, TRPE, ARGD, ACNB, LEUB, FBAB, SDAA, LEUD | 1.226373626 |
|  | stm00400 | Phenylalanine, tyrosine and tryptophan biosynthesis | 3 | 0.423693886 | TRPC, TRPB, TRPE | 2.079503106 |
|  | stm01200 | Carbon metabolism | 9 | 0.423870177 | NIFJ, SUCC, FRDD, ACNB, FBAB, YBHE, SDAA, FUMA, FOLD | 1.247701863 |
|  | stm00240 | Pyrimidine metabolism | 5 | 0.435298794 | USHA, DNAE, DEOD, NRDE, DEOA | 1.476190476 |
|  | stm01120 | Microbial metabolism in diverse environments | 17 | 0.470633997 | ARGD, FRDD, TTRC, RHAA, YBHE, FBAB, FUMA, YADI, NIFJ, SUCC, PHSC, ACNB, ASRA, DLD, HPAB, PGM, FOLD | 1.097281666 |
|  | stm00052 | Galactose metabolism | 3 | 0.547298971 | MALZ, GALT, PGM | 1.649261084 |
|  | stm00670 | One carbon pool by folate | 2 | 0.566960832 | FOLA, FOLD | 2.452747253 |
|  | stm00620 | Pyruvate metabolism | 4 | 0.688728922 | NIFJ, FRDD, DLD, FUMA | 1.13877551 |
|  | stm00053 | Ascorbate and aldarate metabolism | 2 | 0.706384829 | LYXK, YADI | 1.678195489 |
|  | stm00920 | Sulfur metabolism | 3 | 0.709810607 | PHSC, ASRA, TTRC | 1.226373626 |
|  | stm00290 | Valine, leucine and isoleucine biosynthesis | 2 | 0.724835571 | LEUB, LEUD | 1.594285714 |
|  | stm00030 | Pentose phosphate pathway | 3 | 0.748101456 | FBAB, YBHE, PGM | 1.13877551 |
|  | stm00330 | Arginine and proline metabolism | 2 | 0.75836099 | SPEA, ARGD | 1.449350649 |
|  | stm00190 | Oxidative phosphorylation | 3 | 0.75987736 | NUOE, FRDD, NUON | 1.112292359 |
|  | stm00051 | Fructose and mannose metabolism | 3 | 0.811910994 | RHAA, FBAB, YAJF | 0.996428571 |
|  | stm01130 | Biosynthesis of antibiotics | 11 | 0.83249371 | GLMU, SUCC, ARGD, FRDD, ACNB, FBAB, YBHE, SDAA, FUMA, PURD, PGM | 0.863898663 |
|  | stm00260 | Glycine, serine and threonine metabolism | 2 | 0.896563439 | TRPB, SDAA | 0.911020408 |
|  | stm02040 | Flagellar assembly | 2 | 0.90927751 | FLIF, FLHB | 0.861776062 |
|  | stm00010 | Glycolysis / Gluconeogenesis | 2 | 0.920441992 | FBAB, PGM | 0.817582418 |
|  | stm00860 | Porphyrin and chlorophyll metabolism | 2 | 0.920441992 | CBID, CBIF | 0.817582418 |
|  | stm00970 | Aminoacyl-tRNA biosynthesis | 4 | 0.975147098 | PHET, SELA, GLNS, ILES | 0.574517375 |
|  | stm02060 | Phosphotransferase system (PTS) | 2 | 0.977339713 | PTSP, YADI | 0.549753695 |
|  | stm00750 | Vitamin B6 metabolism | 2 | 0.474387599 | PDXA, STM0163 | 3.188571429 |
